# Multi-modal brain magnetic resonance imaging database covering marmosets with a wide age range

**DOI:** 10.1038/s41597-023-02121-2

**Published:** 2023-04-27

**Authors:** Junichi Hata, Ken Nakae, Hiromichi Tsukada, Alexander Woodward, Yawara Haga, Mayu Iida, Akiko Uematsu, Fumiko Seki, Noritaka Ichinohe, Rui Gong, Takaaki Kaneko, Daisuke Yoshimaru, Akiya Watakabe, Hiroshi Abe, Toshiki Tani, Hiro Taiyo Hamda, Carlos Enrique Gutierrez, Henrik Skibbe, Masahide Maeda, Frederic Papazian, Kei Hagiya, Noriyuki Kishi, Shin Ishii, Kenji Doya, Tomomi Shimogori, Tetsuo Yamamori, Keiji Tanaka, Hirotaka James Okano, Hideyuki Okano

**Affiliations:** 1 Graduate School of Human Health Sciences, Tokyo Metropolitan University, Tokyo, Japan; 2 Laboratory for Marmoset Neural Architecture, RIKEN Center for Brain Science, Saitama, Japan; 3 Department of Physiology, Keio University School of Medicine, Tokyo, Japan; 4 Live Animal Imaging Center, Central Institute for Experimental Animals, Kanagawa, Japan; 5 Division of Regenerative Medicine, The Jikei University School of Medicine, Tokyo, Japan; 6 Exploratory Research Center on Life and Living Systems, National Institutes of Natural Sciences, Aichi, Japan; 7 Graduate School of Informatics, Kyoto University, Kyoto, Japan; 8 Center for Mathematical Science and Artificial Intelligence, Chubu University, Aichi, Japan; 9 Neural Computation Unit, Okinawa Institute of Science and Technology Graduate University, Okinawa, Japan; 10 Connectome Analysis Unit, RIKEN Center for Brain Science, Saitama, Japan; 11 Department of Ultrastructural Research, National Institute of Neuroscience, National Center of Neurology and Psychiatry, Tokyo, Japan; 12 Center for the Evolutionary Origins of Human Behavior, Kyoto University, Aichi, Japan; 13 Laboratory for Molecular Analysis of Higher Brain Function, RIKEN Center for Brain Science, Saitama, Japan; 14 Research & Development Department, Araya Inc, Tokyo, Japan; 15 Brain Image Analysis Unit, RIKEN Center for Brain Science, Saitama, Japan; 16 Laboratory for Molecular Mechanisms of Brain Development, RIKEN Center for Brain Science, Saitama, Japan; 17 Laboratory of Haptic Perception and Cognitive Physiology, RIKEN Center for Brain Science, Saitama, Japan; 18 Department of Marmoset Biology and Medicine, Central Institute for Experimental Animals, Kanagawa, Japan

**Keywords:** Neural circuits, Brain

## Abstract

Magnetic resonance imaging (MRI) is a non-invasive neuroimaging technique that is useful for identifying normal developmental and aging processes and for data sharing. Marmosets have a relatively shorter life expectancy than other primates, including humans, because they grow and age faster. Therefore, the common marmoset model is effective in aging research. The current study investigated the aging process of the marmoset brain and provided an open MRI database of marmosets across a wide age range. The Brain/MINDS Marmoset Brain MRI Dataset contains brain MRI information from 216 marmosets ranging in age from 1 and 10 years. At the time of its release, it is the largest public dataset in the world. It also includes multi-contrast MRI images. In addition, 91 of 216 animals have corresponding high-resolution *ex vivo* MRI datasets. Our MRI database, available at the Brain/MINDS Data Portal, might help to understand the effects of various factors, such as age, sex, body size, and fixation, on the brain. It can also contribute to and accelerate brain science studies worldwide.

## Background & Summary

Aging is associated with a decline in brain function, including cognitive function. Magnetic resonance imaging (MRI) databases of the brain have been published for humans of different ages. Integration of these databases has revealed changes in average brain volume with age (e.g., Brain Chart). Changes in the normal aging brain can lead to the development of diseases, such as Alzheimer’s and Parkinson’s disease, and thus provide a better understanding of aging and diseases^1^.

The common marmoset (*Callithrix jacchus*) is a useful non-human primate model of aging^2–4^. It has therefore received considerable attention. Compared to rodents, marmosets have a relatively more similar brain structure to humans^5^ (e.g., layer 4 in the frontal cortex, presence of cytoarchitectural regions not present in rodents, fronto-parietal connectivity more similar to humans^6^). Therefore, they are a more appropriate preclinical animal model for diseases established by drug administration^7^ and/or genetic manipulation^8^. In addition, these animals have a relatively shorter life expectancy (about 10 years in captivity^9^) than other primates, including humans. This makes the aging process easier to follow. Previous studies have examined and published MRI datasets of developmental stages or early adulthood in normal marmosets. Seki *et al*. (2017) provided open-access structural brain datasets (T1-weighted [T1w] and T2-weighted [T2w]; 0–2 years old)^10^ and Uematsu *et al*. (2017) provided open-access diffusion MRI datasets at developmental stage (1–18-month old)^11^. Schaeffer (2022) provides the function MRI dataset of the adult marmoset (14–115 months)^12^ https://www.marmosetbrainconnectome.org/. Liu *et al*. (2020, 2021) published multi-modal, high-quality datasets (T1w, T2w, diffusion-weighted imaging [DWI], awake, anesthetized resting-state functional MRI [rsfMRI]; 3–4 years)^13,14^ (https://marmosetbrainmapping.org/). However, it has not yet covered the wide-range life span, including middle (5–7 years) and late (8–10 years) adulthood of multi-contrast MRI dataset. To identify the aging process of the marmoset brain, we provided an open-accessible MRI dataset covering the wider range life stage of the common marmoset.

The current database contains multi-modal brain MRI datasets on 216 marmosets aged 1–10 years in both *in vivo* and postmortem studies. The male-to-female ratio in the dataset is 4:6, and data on the weight of the animals at each measurement were also available. Thus, the effects of sex and body size on the brain could be examined. All 216 datasets contain T1w and T2w images, and 126 datasets have two-shell *in vivo* DWI images. In addition, 31 datasets have 10-min anesthetized rsfMRI images, and 3 have 20-min awake rsfMRI images (total: 60 min). Furthermore, 91 animals underwent postmortem scans with T2w and DWI images. To our knowledge, this is the largest MRI database to date. Multimodal datasets, including high-resolution postmortem data, could provide not only detailed reliable structural and functional data and its connectivity information but also essential details on the effects of aging on the brain. This openly-accessible database can make a significant contribution to the brain science community.

This database can deepen our understanding of the effects of various factors that affect the brain. For example, the volume of gray matter has been found to decrease with age. In addition, the DWI structural connectivity showed that most of the connectivity peaked at about 3–4 years of age. Furthermore, the strength of the connections was lower in the anesthesized state than in the awake state. These findings are consistent with known human facts, and they support the validity of the marmoset model of development and aging.

## Methods

### Animals *in vivo*

The study included 216 healthy common marmosets (88 male and 128 female, mean weight: 357.1 ± 60.2 g) aged 0.8–10.3 (mean: 4.34 ± 2.56) years. Healthy marmosets were selected from individuals without weight loss or viral infection in the previous two weeks. The common marmosets were anesthetized, and their heads were immobilized prior to imaging. The *in vivo* MRI scan was performed with each animal in the supine position on an imaging table under anesthesia with 2.0% isoflurane (Abbott Laboratories, Abbott Park, IL, the USA) in an oxygen-air mixture. Heart rate, SpO2, and rectal temperature were monitored regularly during imaging to manage the physical condition of the animals. This study was approved by the Animal Experiment Committees of the RIKEN Center for Brain Science (CBS) and conducted in accordance with the Guidelines for Conducting Animal Experiments of RIKEN CBS.

### *In vivo* image acquisition

The marmoset brain MRI dataset (NA216) contains multimodal neuroimaging data including *in vivo* T1w, T2w, DWI, and rsfMRI images. MRI was performed using a 9.4-T BioSpec 94/30 unit (Bruker Optik GmbH, Ettlingen, Germany) and a transmit and receive coil with an inner diameter of 86 mm. For T1w imaging, a magnetization-prepared rapid gradient echo (MP-RAGE) was used, with the following parameters: repetition time (TR) = 6000 ms, echo time (TE) = 2 ms, flip angle = 12°, number of averages (NA) = 1, inversion time = 1600 ms, voxel size = 270 × 270 × 540 μm, and scan time = 20 min. For T2w imaging, rapid acquisition with relaxation enhancement (RARE) was used with the following parameters: TR = 4000 ms, TE = 22 ms, RARE factor = 4, flip angle = 90°, NA = 1, voxel size = 270 × 270 × 540 μm, and scan time = 7 min, 24 s. For DWI, spin-echo echo-planar imaging was used, with the following parameters: TR = 3000 ms, TE = 25.6 ms, δ = 6 ms, Δ = 12 ms, b-value = 1000 and 3000 s/mm^2^ in 30 and 60 diffusion directions, respectively (plus 4 b0 images), number of segments = 6, flip angle = 90°, NA = 3, voxel size = 350 × 350 × 700 μm, and scan time = 90 min. Diffusion metrics were generated using the diffusion tensor imaging (DTI) model, and the diffusion fiber connectome was generated using constrained spherical deconvolution^15^. For rsfMRI, gradient-recalled echo-planar imaging was used, with the following parameters: TR = 1500 ms, TE = 18 ms, number of shots = 1, flip angle = 40°, NA = 1, number of repetitions = 400, voxel size = 500 × 500 × 1000 μm, and scan time = 10 min.

### Treatment of *ex vivo* imaging animals

Each animal was perfusion-fixed with 4% paraformaldehyde (PFA), and the brain was dissected from the skull and immersed in PFA for *ex vivo* imaging. During *ex vivo* imaging, the brain was wrapped in a sponge and soaked in fluorine solution, which does not show signal on MRI images, in a plastic container. Vacuum degassing was performed to reduce artifacts. The PFA solution used for fixation was replaced with fresh solution weekly to maintain fixation.

### *Ex vivo* acquisition

MRI was performed using a 9.4-T BioSpec 94/30 unit (Bruker Optik GmbH) and a transmit and receive solenoid type coil with inner diameter of 28 mm. RARE was used for T2w imaging with the following parameters: TR = 10,000 ms, TE = 29.3 ms, RARE factor = 4, flip angle = 90°, NA = 16, voxel size = 100 × 100 × 200 μm, and scan time = 3 h, 20 min. For DWI, spin-echo echo-planar imaging was used, with the following parameters: TR = 4000 ms, TE = 28.4 ms, δ = 7 ms, Δ = 14 ms, b-value = 1,000, 3,000, and 5,000 s/mm^2^ in each of 128 diffusion directions (plus 6 b0 images), number of segments = 10, flip angle = 90°, NA = 2, voxel size = 200 × 200 × 200 μm, and scan time = 6 h, 39 mins.

### Data processing pipeline

#### Structural image

**Fig. 1:**
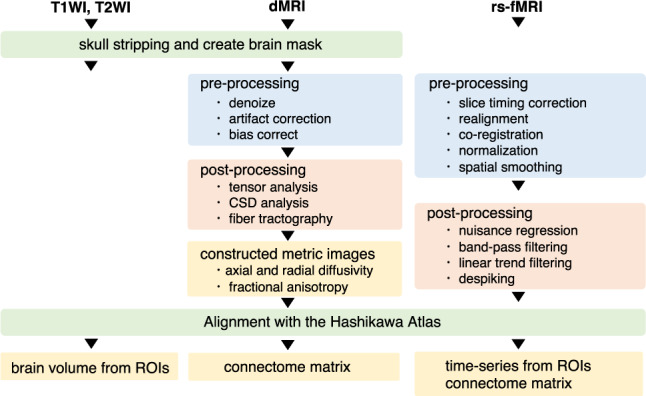
Schematics of the processing of our pipeline from T1WI, T2WI, dMRI and rs-fMRI.

The schematic of the processing pipeline for structural, diffusion and function MRI is shown in Fig. 1. To correct T2w images, whole brains were extracted from the image data using BrainSuite18a (David W. Shattuck, Ahmanson-Lovelace Brain Mapping Center at the University of California). Mask images were generated and a registration process was performed to align the standard brain images by mapping brain region data to the structural images of each animal. The analysis software ANTs (Brian B. Avants, University of Pennsylvania) was used for this process^16^.

To locate brain regions, we digitized the Atlas^17^ proposed by Hashikawa *et al*.^18^ in a 3D setting. Since the Hashikawa atlas was segmented by histology, whose resolution scale is extremely high for MRI data analysis, we merged the regional labels into 6 and 52 and 111 anatomically validated regions defined by the anatomist, which are suitable for both structural and functional MRI analysis.

The migration information from the standard brain image to the structural image of each animal was calculated. This information was superimposed on the brain region data to generate information corresponding to the structural image of each animal. The T1w/T2w approach was proposed by Glasser *et al*. in 2011, and it showed how to increase the contrast related to myelin content by calculating a simple ratio between T1w and T2w images^19^. Since this can be calculated from the ratio of T1w and T2w images, there was no need for novel imaging^20^.

#### Diffusion MRI

Pre-processing steps, such as artifact removal, were performed. These processes were performed using the brain image analysis tool Mrtrix3 version 3.0.3.12 (J-Donald Tournier, School of Biomedical Engineering & Imaging Sciences, King’s College London)^21^. The following commands were used in various processes: dwidenoize, mrdegibbs, dwipreproc, and dwibiascorrect. After image pre-processing, diffusion metrics were created using the DTI model. The diffusion fiber connectome was created using constrained spherical deconvolution (Tournier *et al*., 2004), and the *ex vivo* diffusion fiber connectome was created using high angular resolution diffusion-weighted MRI^22^. We used the MRTrix3 software for tensor analysis and fiber construction (dwi2tensor, tensor2metrics, dwi2response, dwi2fod, taken, and SIFT). We constructed diffusion metric images, axial diffusivity (AD) images, radial diffusivity (RD) images, fractional anisotropy (FA) images, and connectome matrices based on the number of fibers.

#### Resting-state functional imaging

Data pre-processing was performed using the SPM12 software package (Wellcome Department of Cognitive Neurology, London, UK) running under MATLAB (MathWorks, Natick, MA, USA). We then performed denoising steps using the functional connectivity toolbox (CONN). The empirical blood oxygenation level-dependent signals were band-pass filtered within a narrow band of 0.01–0.08 Hz. The analysis used fMRI data from a 20-min scan (initial 40 volumes discarded; subsequent 560 functional volumes) for awake data and a 10-min scan (initial 20 volumes discarded; subsequent 380 functional volumes) for anesthetized data. The empirical FC matrix was calculated using Pearson correlation between the average time courses of 104 brain regions for 3 awake and 31 anesthetized healthy common marmosets at rest and was averaged across the marmosets.

#### Brain regions

In this study, we used the anatomically segmented atlas of the common marmoset brain reported by Hashikawa *et al*.^18^, The atlas was applied to the data from 111 regions of one brain and 52 regions of another brain created by combining several of the 111 regions. The regions of interest (ROIs) are listed in ROImerge_data_v2.xlsx. In addition, the data were divided into six regions (cerebrospinal fluid, gray matter, deep gray matter, white matter, cerebellum, and brainstem) were obtained for large-scale segmentation, and were fitted to individual brain data. ANTs software was used to align the brain atlas to the individual brains.

## Data Records

All datasets are publicly available on the Brain/MINDS Data Portal (10.24475/bminds.mri.thj.4624)^23^. The dataset is divided into four sections, which are as follows: *in vivo* MRI from 216 animals, *ex vivo* MRI from 91 animals, standard brain, and BMA 2019 atlas mappings.

**Fig. 2:**
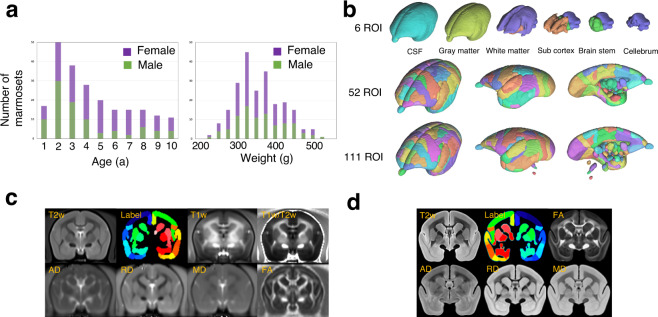
Summary of the marmoset magnetic resonance imaging (MRI). (**a**) Histograms of age and weight of marmosets according by sex. (**b**) Multiscale label map with a three-dimensional visualization of the merged regions of the Hashikawa atlas into 6, 52, and 111 regions of interest (ROIs). (**c**) For *in vivo* MRI data, T2-weighted (T2w), label, T1-weighted (T1w), T1w-to-T2w ratio, axial diffusivity (AD), radial diffusivity (RD), mean diffusivity (MD), and fractional anisotropy (FA) images are shown. (**d**) For *ex vivo* MRI data, T2w, label, FA, AD, RD, and MD images are shown.

The *in vivo* MRI metadata are described in Individual_information_invivo.xlsx. The metadata includes the following information for each of the 216 animals: ID, age, sex, weight, relaxometry image, diffusion MRI image and structural connectome, label image, and anesthesia (as shown in Fig. 2). The *in vivo* MRI data set includes the following: T1w image (T1WI_*.nii.gz), T2w image (T2WI_*.nii.gz), myelin contrast image (T1wT2w_*.nii.gz), diffusion weighted image (i_dwi_*.nii) with b-value and b-vectors (i_DWI_MPG_info.zip), AD image (dtiAD_*.nii.gz), RD image (dtiRD_*.nii.gz), mean diffusivity (MD) image (dtiMD_*.nii.gz), FA image (dtiFA_*.nii.gz), FA color image (dtiFAc_*.nii.gz), anesthetized resting state fMRI image on an original space (fmri_aneth_raw_*.nii.gz) and on a standard space (fmri_aneth_deformed_*.nii.gz), awake resting state fMRI image on an original space (fmri_awake_raw_*.nii.gz) and on a standard space (fmri_awake_deformed_*.nii.gz), 6 ROI label map image (label006_*.nii.gz), 52 ROI label map image (label052_*.nii.gz), and 111 ROI label map image (label111_*.nii.gz). The deformation fields of ANTs format from the original space to the standard space is defined (i_deformable_info.zip). These can be downloaded in NIFTI format (nii.gz) for each individual marmoset. In addition, the volume for each ROI of the BMA 2019 atlas (Brain_*_summary.xlsx, within i_Variables_gm.zip, with the atlas available at 10.24475/bma.4520)^24^, structural connectome between ROIs (DiffusionSC_*.csv), anesthetized functional connectome (AnethFC_*.csv), and awake functional connectome (AwakeFC_*.csv) can be downloaded for each individual.

The *ex vivo* MRI data are described in Individual_information_exvivo.xlsx. The metadata include ID, age, sex, weight, T2w image, diffusion MRI image and structural connectome, label image, and ID of the corresponding *in vivo* MRI data for each of the 91 animals. The *ex vivo* MRI dataset includes the following: T2w image (T2Wi_ex*.nii.gz), diffusion weighted image (dwi_ex*.nii) with b-value and b-vectors (e_DWI_MPG_info.zip), AD image (dtiAD_ex*.nii.gz), RD image (dtiRD_ex*.nii.gz), MD image (dtiMD_ex*.nii.gz), FA image (dtiFA_ex*.nii.gz), FA color image (dtiFAc_ex*.nii.gz), and 52 ROI label map image (label052_ex*.nii.gz). The deformation field of ANTs format from the original space to the standard space is defined (e_deformable_info.zip). These can be downloaded in NIFTI format for each individual. In addition, the volume of each ROI (Brain_ex*_summary.xlsx, within e_Variables_gm.zip) for the BMA 2019 atlas and the structural connectome (DiffusionSC_*ex*.csv) between the ROIs of each value can be downloaded for each individual.

The standard brain section was averaged the images after the registration of the individual data to the BMA 2019 atlas. The BMA 2019 atlas mapping section provides data on the BMA 2019 atlas mapped to the *in vivo*, *ex vivo*, and standard spaces of each individual brain.

## Technical Validation

### Brain volume

**Fig. 3:**
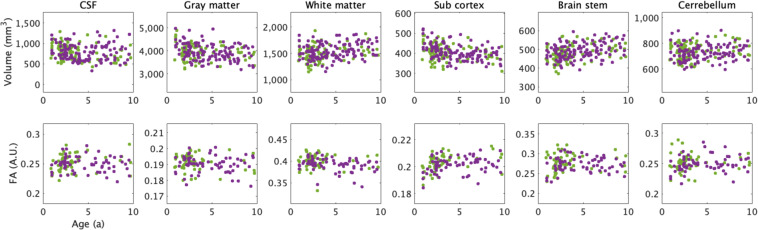
Scatter plot of volume size and mean fractional anisotropy (FA) values versus age for marmosets. The purple dots correspond to female marmosets, while the green dots correspond to male marmosets. The upper panels show the volume of the cerebrospinal fluid (CSF), cortex, white matter, subcortex, brainstem, and cerebellum as a function of age. The lower panels show the FA of the CSF, cortex, white matter, subcortex, brainstem, and cerebellum as a function of age.

We evaluated age-related volumetric changes in each brain region by analyzing T2w images, as shown in Fig. 3. The volume of the gray and white matter decreased from the developmental age (approximately 12 months) to maturity (approximately 18 months), followed by a gradual downward trend, similar to that observed in humans aged 20–50 years in a similar study^25^. In addition, a gradual decrease in the cortical volume of common marmosets was consistent with that of a previous study^7^ that assessed the common marmoset brain volume from 1 month to 18 months of age. Despite the overall decrease in brain volume with age, the proportion of white matter increases with age. In humans, cerebrospinal fluid volume increases significantly as the brain atrophies with old age^26^. However, no significant increase was observed in this study. Therefore, caution should be used in comparative studies of age-related cerebrospinal fluid changes involving humans or other non-human primates. An increase in individual variability of the brain volume was observed with age, and the age-related variance values were 19.27%, 25.18%, and 28.75% for the ages 18–36 months, 37–72 months, and ≥73 months, respectively.

### Diffusion-weighted structural MRI

The mean values of the DTI metrics, including FA, AD, and RDr in each brain region, were evaluated and summarized as a scatter plot to confirm the data distribution. We observed that FA in the white matter of common marmosets had a slightly higher dispersion than AD and RD in the white matter of marmosets. A large variation in the white matter tissue was observed with different values based on the frontal and posterior areas; the data showed a decreasing trend with age. When compared with previous human studies, the FA was consistent with a decline up to 40 years of age in human studies^27^. In the deep gray and white matter, FA increases up to 4 years of age, and it does not change significantly thereafter. However, no changes were observed in regions other than the deep gray matter. In humans, the number increases until 20 years of age and does not change significantly thereafter. It then declines at about 40 years of age^28^. In both common marmosets and humans, FA is lower than the average FA for all ages up to the developmental period. This suggests that myelin development limits intracellular diffusion until development is complete and that FA stabilizes after development. The decrease in diffusion coefficient with age is related to the fact that myelination limits proton diffusion in the extracellular space as cells develop^29,30^.

**Fig. 4:**
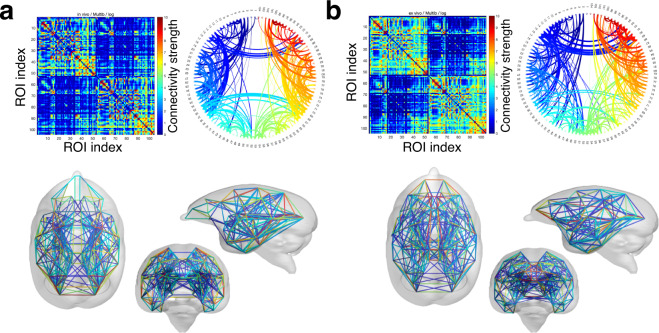
Mean values of structural connectivity estimated from diffusion magnetic resonance imaging (dMRI). (**a**) *In vivo* dMRI: The top left panel shows the mean of the connectivity matrix calculated from tractography for 216 marmosets. The upper right panel shows the circular connectivity plot with the connections colored according to region, and the lower panel depicts the connectivity plot from the centroid of gravity of the region of interest (ROI) location according to region. (**b**) *Ex vivo* dMRI: The upper left panel shows the mean of the connectivity matrix of 91 marmosets calculated from tractography. The upper right panel shows the circular connectivity plot with the connections colored according to region, and the lower panel shows the connectivity plot from the centroid of gravity of the ROI location according to region.

Figure 4 shows the whole brain connectivity derived from diffusion tractography. Our connectivity matrices in *in vivo* and *ex vivo* datasets showed that connections are stronger on the ipsilateral side and weaker in the contralateral brain. When the distance between brain regions is greater, the number of connections in the neural structure network on diffusion MRI is less. The connection trends between the brain regions are almost similar between the *in vivo* and *ex vivo* connections, and the strength was more significant in the *ex vivo* data, which indicated that the time was longer in the *ex vivo* data. Although there are few differences, large diffusion fiber connectivity is observed in both *in vivo* and *ex vivo* data.

### Resting-state functional MRI

**Fig. 5:**
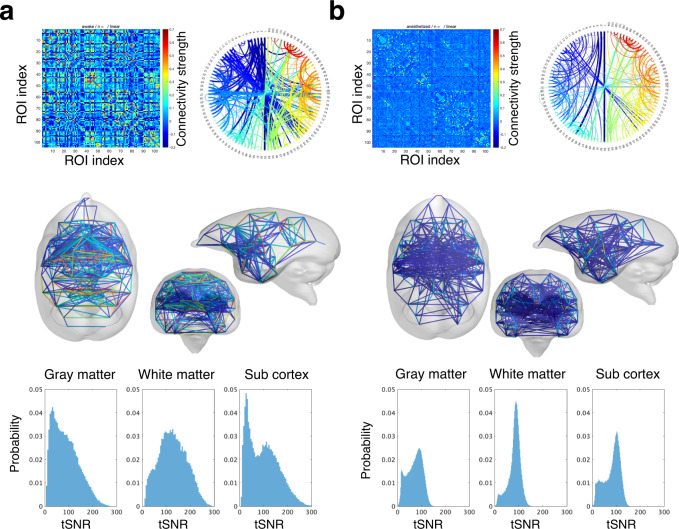
Mean values of functional connectivity estimated from functional magnetic resonance imaging (fMRI). (**a**) Waking fMRI: The upper left panel shows the mean of the cross-correlation matrix calculated from fMRI activity for three marmosets. The upper right panel shows the circular connectivity plot with connections colored by region, and the middle panel shows the connectivity plot from the center of gravity of the region of interest (ROI) location based on region. The lower panel shows the histogram of temporal SNR (tSNR) of the registered fMRI voxels in the regions of gray matter, white matter, and subcortical regions. (**b**) Anesthetized fMRI: The upper left panel shows the mean of the cross-correlation matrix of 31 marmosets calculated from the fMRI activity. The upper right panel shows the circular connectivity plot with the connections colored by region, and the middle panel shows the connectivity plot from the center of gravity of the ROI location based on region. The lower panel shows the histogram of the temporal SNR (tSNR) of the registered fMRI voxels in the regions of gray matter, white matter, and subcortical regions.

The actual brain activities in the resting condition are difficult to be directly evaluated, but recent techniques have made it possible to evaluate awake rsfMRI available^31–33^. Head implant surgery is performed under general anesthesia followed by analgesia to avoid pain, and animals are habituated to head restraints for several weeks, which reduces stress. The detailed condition of awake and anesthetized training and restraint with head movement data is described in Muta *et al*. (2022)^33^. Under anesthesia, the BOLD signal of rsfMRI is low compared to the awake state, as shown in Fig. 5. The current dataset includes data obtained under both anesthesia and wakefulness. Therefore, the level of brain activity differed between the anesthetized and awake states (as shown in Fig. 5). The temporal SNR of fMRI voxels from all marmosets in the regions of gray matter, white matter, and subcortical regions are shown in the lower panels of Fig. 5. The tSNR of the anesthetized data is lower than that of the tSNR of the awake data.

## Data Availability

Brain/MINDS Data portal^23^, BrainSuite18a^34^, ANTs (Advanced Normalization Tools)^16^, Mrtrix3^21^, SPM12 (Statistical Parametric Mapping package)^35^, CONN (the functional connectivity toolbox)^36^.
